# Dexmedetomidine enhances tolerance to bupivacaine cardiotoxicity in the isolated rat hearts: alpha 2 adrenoceptors were not involved

**DOI:** 10.1186/s40360-019-0371-1

**Published:** 2019-11-21

**Authors:** Fangfang Xia, Zhousheng Jin, Tingting Lin, Xixi Cai, Linmin Pan, Shi Wang, Yaoyao Cai, Hongfei Chen

**Affiliations:** Department of Anesthesiology, The First Affiliated Hospital of Wenzhou Medical University, Shangcai village, Nanbaixiang town, Ouhai District, Wenzhou City, 325000 Zhejiang Province China

**Keywords:** Dexmedetomidine, Bupivacaine, Cardiotoxicity, Alpha 2 adrenoceptors, Isolated heart

## Abstract

**Background:**

Dexmedetomidine was proved to mitigate bupivacaine-induced cardiotoxicity but mechanism of this ability is still unclear. This study was designed to investigate the direct effects of dexmedetomidine on cardiotoxicity induced by bupivacaine on Langendorff rat heart preparation and the role of alpha 2 adrenoceptors in this process was explored.

**Methods:**

Hearts of rat were isolated, mounted on a Langendorff system. Five experimental groups were assessed after 10 min Krebs-Henseleit buffer (KHB) infusions as follow: (1) Group Con, only KHB was perfused; (2) Group Dex, KHB was perfused for 5 min, then dexmedetomidine (10 nmol/L) was added; (3) Group Bupi, KHB was perfused for 25 min, then bupivacaine (50 μmol/L) was added; (4) Group Bupi + Dex, KHB was perfused for 5 min, then the dexmedetomidine (10 nmol/L) was added for 20 min, at last a mixture of KHB + dexmedetomidine + bupivacaine were perfused; (5) Group Bupi + Dex + Yoh, a combination of KHB + yohimbine (alpha 2 adrenoceptor antagonists, 1 μmol/L) was perfusion for 5 min, then dexmedetomidine (10 nmol/L) was added for 20 min, at last a mixture of KHB + yohimbine + dexmedetomidine + bupivacaine was perfused. The experimental perfusion was maintained for 35 min in group Con and group Dex, and the experimental perfusion was sustained until asystole in the other three groups.

**Results:**

Compared with group Bupi, dexmedetomidine significantly increased the time to first arrhythmia (*P* <  0.001) and time to asystole (*P* <  0.001) in group Bupi + Dex. In addition, dexmedetomidine also significantly increased the time to 25, 50 and 75% reductions in heart rate (*P* <  0.001) and the time to 25, 50 and 75% reductions in rate-pressure product (*P* <  0.001) in group Bupi + Dex. Dexmedetomidine increased the cardiac tissue bupivacaine content when asystole (Bupi + Dex vs. Bupi, 58.5 ± 6.3 vs. 46.8 ± 5.6 nmol/g, *P* = 0.003). The benefit of dexmedetomidine on bupivacaine-induced cardiotoxicity were not eliminated by yohimbine.

**Conclusions:**

Dexmedetomidine could delay the occurrence of bupivacaine-induced arrhythmia and asystole in the isolated rat hearts, but the alpha 2 adrenoceptors were not involved in this process.

## Background

Bupivacaine is a long-acting, lipophilic, amide class local anesthetic commonly used to provide local anesthesia during surgical procedures in clinical practice because of its high analgesic potency and long-lasting effects. Unfortunately, bupivacaine exhibits a strong tendency to cardiotoxicity when unintentional intravenous injection or overdose [1]. Cardiotoxicity induced by bupivacaine, which can lead to arrhythmias, poor myocardial contractility and even cardiac arrest, is difficult to reverse [2].

Dexmedetomidine, a highly selective α-2 adrenoceptor agonist with a broad range of pharmacological properties, which is widely used for its sedative effects clinically [3, 4]. Accumulating evidence suggests that dexmedetomidine could be used as a local anesthetics adjuvant to enhance the analgesic effect [5–7], and also provide sedation [5]. Hanci et al. [8] reported dexmedetomidine pretreatment could strengthen tolerance to bupivacaine-induced cardiotoxicity in rats. It demonstrated dexmedetomidine (10 μg/kg) pretreatment increased the time to decreases in heart rate, reductions in mean arterial pressure, first arrhythmia and asystole. Hanci suspected that dexmedetomidine’s ability to mitigate bupivacaine-induced cardiotoxicity may be related with its sympatholytic properties [9] and anti arrhythmogenic [10]. However, the action sites of dexmedetomidine mitigate local anesthetics-induced cardiotoxicity has not yet been delineated.

Our primary hypothesis was that dexmedetomidine can mitigate cardiotoxicity of bupivacaine by acting directly on the isolated heart. In this study, we established bupivacaine cardiotoxicity model in the isolated rat hearts and investigated the direct effects of dexmedetomidine on cardiotoxicity induced by bupivacaine. The main indicators in our experiment are the time to the first arrhythmia (T_arrhythmia_) and the time to asystole (T_asystole_), secondary indicators include the cardiac tissue bupivacaine content when asystole.

## Methods

### Animals

All animal protocols were approved by the Wenzhou Medical University Animal Care and Use Committee (wydw 2015–0121, Zhejiang, China). Adult male Sprague-Dawley rats (SYXK 2015–0150), weighing between 320 and 380 g were provided by the Animal Center of Wenzhou Medical University. The care and handling of animals were in accordance with National Institutes of Health guidelines. All animal’s body were incinerated after the study by the Animal Center of Wenzhou Medical University. A completed ARRIVE (Animal Research: Reporting of In Vivo Experiments) guidelines checklist is included in Checklist.

### Drugs

Bupivacaine hydrochloride (0.5%, Sigma-Aldrich Co., St. Louis, MO, P code: 101524503 B5274-5G), Dexmedetomidine (Sigma-Aldrich Co., St. Louis, MO, P code: 12815 SML0956-10MG) and Yohimbine (Sigma-Aldrich Co., St. Louis, MO, P code: 101509939 Y3125-1G) were used.

### Preparation of isolated hearts

The isolated perfused, nonrecirculating Langendorff rat heart preparation was used in our study, as described previously [11]. Rats were anesthetized by the intraperitoneal injection of 80 mg/kg ketamine hydrochloride and 12 mg/kg xylazine, then 1000 U/kg heparin to prevent the formation of intracoronary microthrombi. The rats were euthanized by cervical dislocation, then the hearts were rapidly excised and perfused via the coronary arteries by tying the aorta onto a cannula (ML870B2, AD Instruments, Australia). The constant perfusion pressure was 80 mmHg, and a modified Krebs-Henseleit buffer (KHB) was used and described as follows: NaCl 118 mmol/L, KCl 4.7 mmol/L, MgSO_4_ 1.2 mmol/L, KH_2_PO_4_ 1.2 mmol/L, NaHCO_3_ 25.0 mmol/L, CaCl_2_ 2.5 mmol/L, glucose 10 mmol/L. The solution was continuously bubbled with 95% O_2_ and 5% CO_2_, and pH was maintained at 7.40 ± 0.05. The left ventricular pressure was continuously monitored by a latex balloon placed in the left ventricle. Saline was intermittently injected into the balloon to maintain the left ventricular end-diastolic pressure at 4–10 mmHg. Electrocaridiograph (ECG) electrodes were consistently placed in a “leadII” position. All data were collected using a PowerLab biological signal processing and analysis system (ML870, Australia Ad Instruments) and the Chart 5.5.6 biological signal recording software. The experimental protocol was started after the 10 min of KHB as the steady-state baseline conditions.

### Experimental protocol

**Fig. 1:**
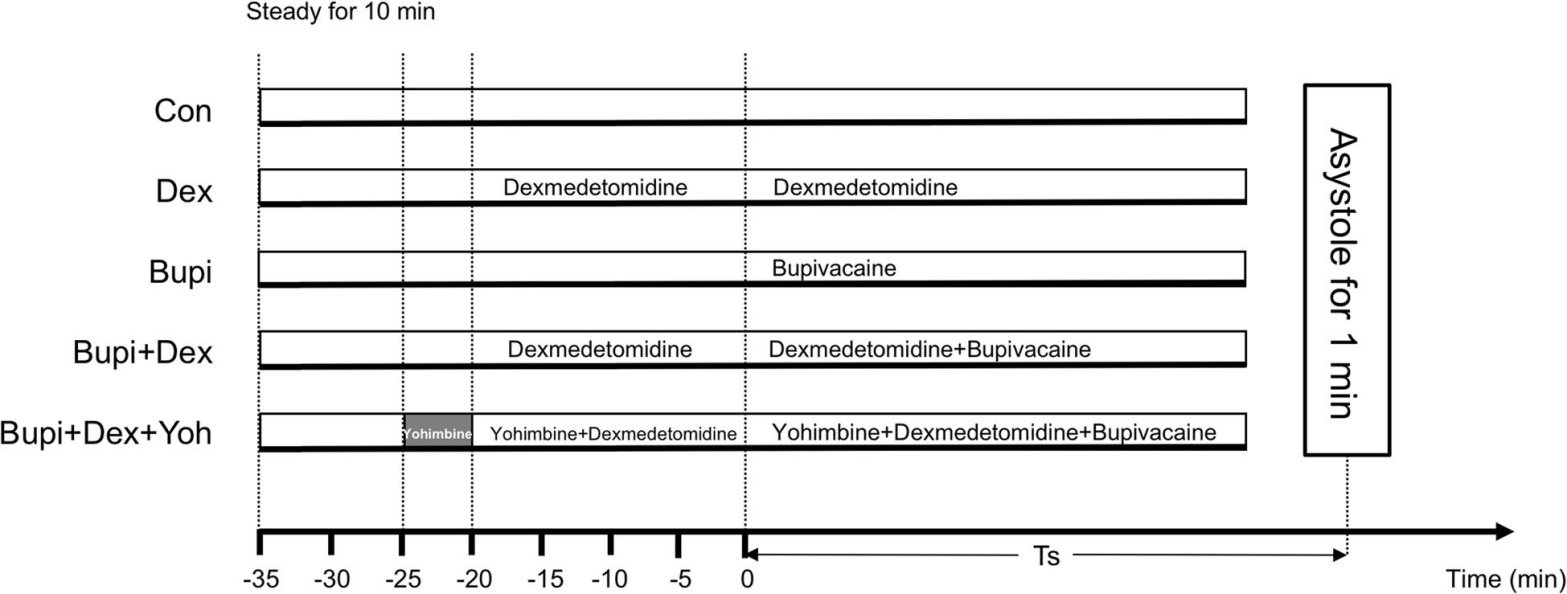
Experimental Protocol (*n* = 8 in each group). Con, control; Bupi, bupivacaine; Dex, dexmedetomidine; Yoh, yohimbine. Without the infusion of bupivacaine, there was no arrhythmia or asystole occurred in group Con and group Dex. As a result, there was no values for T_arrhythmia_, T_asystole_, the time of reductions in HR and RPP in this table

Forty isolated rat hearts were mounted on the Langendorff system and then randomly assigned to 5 groups (Fig. 1): Group Con, Group Dex, Group Bupi, Group Bupi + Dex and Group Bupi + Dex + Yoh (*n* = 8). The KHB was continuously perfused into the hearts in all groups until the end. Experimental perfusion was started according to the assigned group after steady state was reached. In group Con, only KHB was perfused; In group Dex, dexmedetomidine and KHB were perfused; In group Bupi, KHB was perfused 25 min and then 50 μmol/L bupivacaine was added, whereas in group Bupi + Dex, 10 nmol/L dexmedetomidine was added after 5 min of KHB perfusion, and 50 μmol/L bupivacaine was added 20 min later. Besides, in group Bupi + Dex + Yoh, 1 μmol/L yohimbine was added immediately after the steady state, then 10 nmol/L dexmedetomidine was added 5 min later, and 50 μmol/L bupivacaine was added after 20 min later. In all groups but the group Con and group Dex, the experimental perfusion was sustained until asystole, and group Con and group Dex were perfused for 35 min. The recorded data were evaluated and the following times were documented for each heart in all groups: the time of the first premature ventricular contraction accompanied by abnormal systole on the pressure trace after the bupivacaine perfusion (T_arrhythmia_, Time to the first arrhythmia); the time from the initiation of 50 μmol/L bupivacaine infusion to asystole (> 1 min) (T_asystole_, Time to asystole); time to 25, 50 and 75% reductions in heart rate (HR) and rate-pressure product (RPP = HR×(systolic pressure - diastolic pressure)) relative to baseline. Baseline values of hemodynamic were obtained after the 10 min with only KHB perfusion.

### Cardiac tissue bupivacaine content [12]

After asystole, heart apex was cut immediately and stored in liquid nitrogen. Frozen hearts were grinded in a precooling glass tissue grinder with ice and homogenized with 0.4 mol/L perchloric acid by 10 mL/g. Precipitated proteins were separated by centrifugation at 4000 *g* for 15 min, and the supernatant was neutralized with 2 mol/L KOH to adjust pH at 6.0–7.0 by pH meter. Samples were then centrifuged at 4000 *g* for another 15 min and the supernatant was filtered through a 0.22 μm filter to determine the sample. The above operation was completed at 4 °C. The concentration of bupivacaine in cardiac tissue was determined by HPLC-MS/MS (High performance liquid chromatography-mass spectrometry/mass spectrometry).

### Statistical analysis

The power analysis was based on our preliminary study which is mentioned above using the Power Sample Size (PASS 11.0) software program. We compared T_arrhythmia_ among the various treatment groups. In our preliminary study, we totally used 9 rats: 3 rats in each of 3 groups. The means ± standard deviation(SD) of T_arrhythmia_ in Bupi, Bupi + Dex and Bupi + Dex + Yoh groups were 161.0 ± 4.9, 283.7 ± 15.2, and 283.3 ± 5.9, respectively. Using a two-tailed type one error at 5% and type two error of 10%(α = 0.05, β = 0.1), the sample size of 6 per group was obtained. Balancing this fact with the desire to reduce the use of animals, we enrolled 8 rats per group for the study. At this point in the development of an assay sample sizing should only be viewed as a guide because the estimates of both the effect size and associated variability are approximate and come with much uncertainty attached to them.

SPSS (version 19.0, Chicago, IL) was used to carry out the computations. The measurement data were tested for normality using the Shapiro-Wilk test, normally distributed data were presented as the means ± SD. One-way ANOVA was used to evaluate differences among groups, and the Tukey test was used as a post hoc test when significance was achieved. Statistical significance was considered as *P* <  0.05.

## Results

There were no differences in baseline weight (351.1 ± 15.3, 342.9 ± 17.5, 353.5 ± 19.2, 352.3 ± 21.2, 349.1 ± 22.3 g for groups Con, Dex, Bupi, Bupi + Dex and Bup + Dex + Yoh, respectively), baseline HR (300.8 ± 13.6, 297.6 ± 19.2, 296.3 ± 14.0, 298.0 ± 21.3, 299.4 ± 19.8 beats/min, respectively), baseline RPP (36,788.8 ± 2431.2, 36,810.8 ± 2731.6, 36,096.1 ± 1863.2, 36,452.4 ± 2670.2, 37,456.3 ± 3125.5 mmHg·beats/min, respectively) among the 5 groups in the study(*P >* 0.05). No obvious adverse reactions occurred throughout the experiment.

### T_arrhythmia_ and T_asystole_

Table 1 showed that the T_arrhythmia_ in the Bupi + Dex and Bupi + Dex + Yoh groups were comparable (*P* = 0.998), but both were significantly longer than the Bupi group (*P* <  0.001, *P* <  0.001, respectively). Similarly, there was no difference in the T_asystole_ between the Bupi + Dex and Bupi + Dex + Yoh groups (*P* = 0.990), but both T_asystole_ were significantly longer than that in the Bupi group (*P* <  0.001, *P* <  0.001, respectively).

**Table 1:** Times to defined end points in the HR, RPP and ECG after bupivacaine perfusion

Times to event (seconds)	Con	Dex	Bupi	Bupi + Dex	Bupi + Dex + Yoh		*p*
T_arrhythmia_	–	–	160.9 ± 23.5	277.8 ± 27.1	278.1 ± 31.2	Con vs. Bupi*+*Dex	< 0.001
						Con vs. Bupi+Dex Yoh+	< 0.001
						Dex vs. Yoh + Dex	0.100
T_asystole_	–	–	360.3 ± 11.5	546.1 ± 23.8	543.16 ± 37.2	Con vs. Dex	< 0.001
						Con vs. Yoh + Dex	< 0.001
						Dex vs. Yoh + Dex	0.099
HR, 25% reduction	–	–	131.8 ± 14.0	179.8 ± 24.8	180.3 ± 14.8	Con vs. Dex	< 0.001
						Con vs. Yoh + Dex	< 0.001
						Dex vs. Yoh + Dex	0.096
HR, 50% reduction	–	–	257.1 ± 26.6	326.5 ± 23.7	330.6 ± 28.5	Con vs. Dex	< 0.001
						Con vs. Yoh + Dex	< 0.001
						Dex vs. Yoh + Dex	0.097
HR, 75% reduction	–	–	303.1 ± 20.6	418.1 ± 57.0	424.3 ± 27.2	Con vs. Dex	< 0.001
						Con vs. Yoh + Dex	< 0.001
						Dex vs. Yoh + Dex	0.080
RPP, 25% reduction	–	–	121.9 ± 15.8	165.5 ± 14.3	160.6 ± 13.4	Con vs. Dex	< 0.001
						Con vs. Yoh + Dex	< 0.001
						Dex vs. Yoh + Dex	0.099
RPP, 50% reduction	–	–	223.6 ± 25.5	316.4 ± 23.4	321.3 ± 23.7	Con vs. Dex	< 0.001
						Con vs. Yoh + Dex	< 0.001
						Dex vs. Yoh + Dex	0.093
RPP, 75% reduction	–	–	285.4 ± 29.0	412.6 ± 25.1	409.5 ± 27.3	Con vs. Dex	< 0.001
						Con vs. Yoh + Dex	< 0.001
						Dex vs. Yoh + Dex	0.064

Tabel 1. Data were given as mean ± SD. (*n* = 8 in each group)

^*^P < 0.05 versus Group Con. *HR* Heart rate, *RPP* Rate-pressure product, *KHB* Krebs-Henseleit buffer, *Con* Control, *Dex* Dexmedetomidine, *Yoh* Yohimbine

### Cardiac function variables---- HR, RPP

**Fig. 2:**
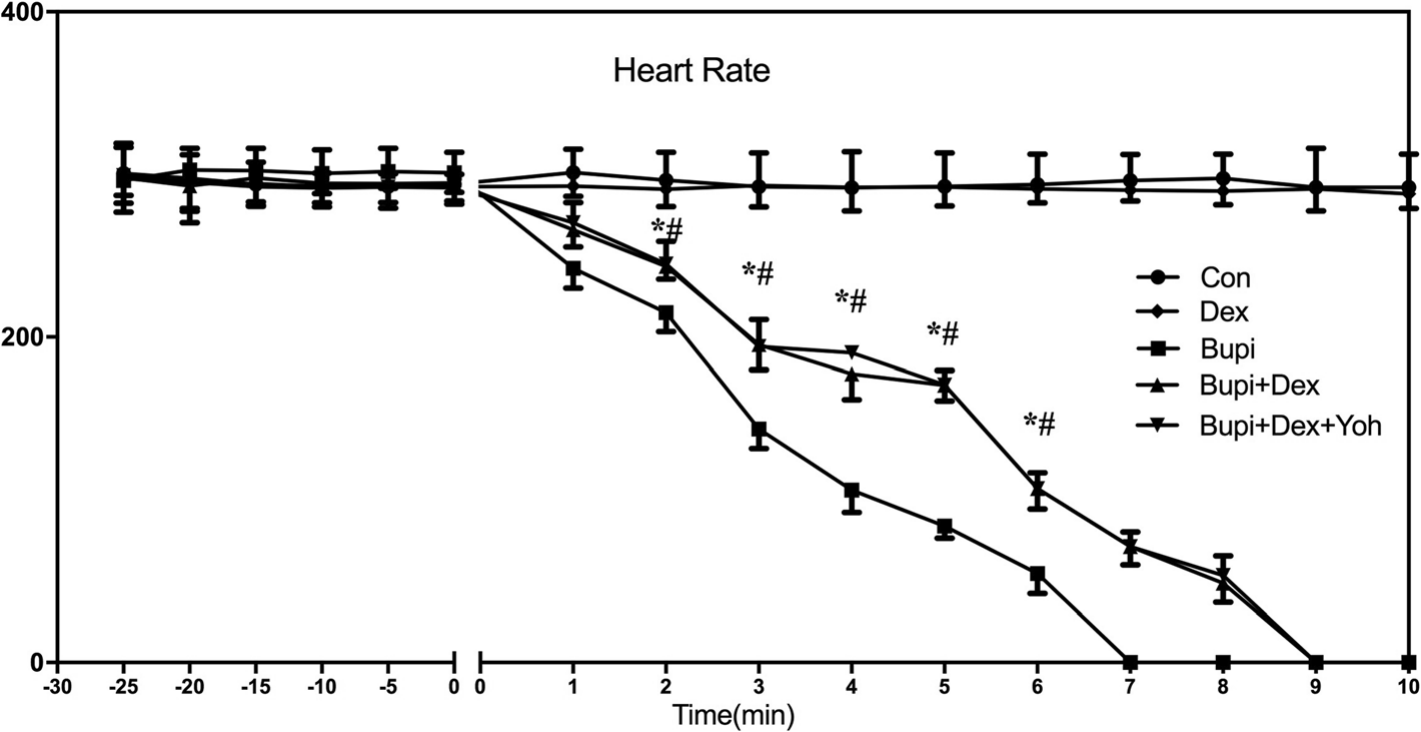
Changes of HRs in five groups (*n* = 8 in each group). HR (Heart Rate) in three groups were presented graphically. Data were given as mean ± SD. ^*^P <  0.05, between group Bupi and group Bupi + Dex, ^#^*P* <  0.05, between group Bupi and group Bupi + Dex + Yoh. Con, control; Bupi, bupivacaine; Dex, dexmedetomidine; Yoh, yohimbine

**Fig. 3:**
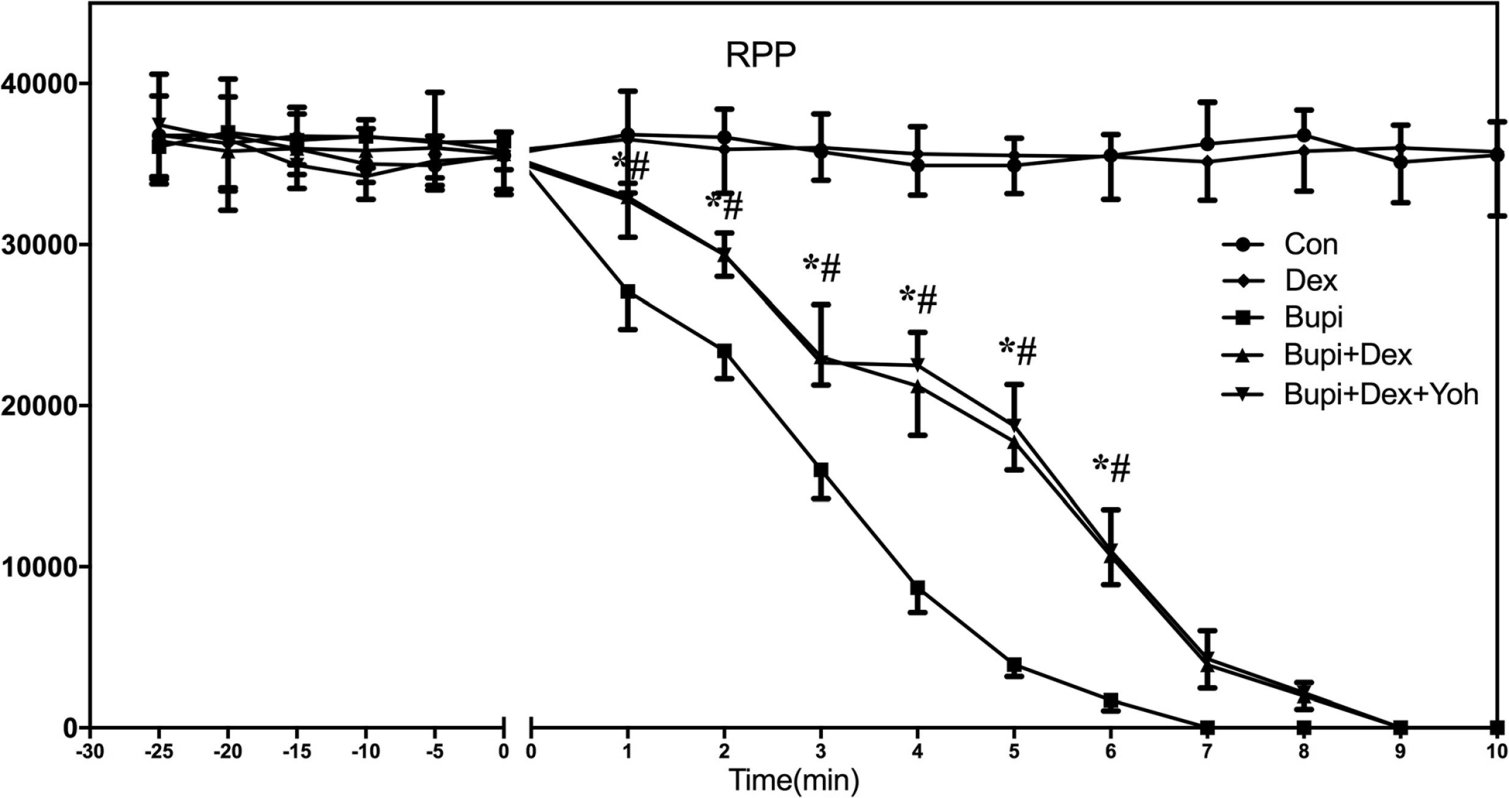
Changes of RPPs in five groups (*n* = 8 in each group). RPP (Rate-pressure product) in three groups were presented graphically. Data were given as mean ± SD. ^*^P <  0.05, between group Bupi and group Bupi + Dex, ^#^*P* <  0.05, between group Bupi and group Bupi + Dex + Yoh. Con, control; Bupi, bupivacaine; Dex, dexmedetomidine; Yoh, yohimbine

The HRs and RPPs in group Con showed no difference with the baseline throughout the experiment (*P* > 0.05). After infusion of dexmedetomidine, the HRs and RPPs also showed no statistical differences between group Dex and group Con (*P* > 0.05). (Figs. 2 and 3).

The time to 25, 50 and 75% reductions in HR and the time to 25, 50 and 75% reductions in RPP were significantly increased in group Bupi + Dex than those in group Bupi (*P* <  0.001 for all comparisons, Table 1). The time to 25, 50 and 75% reductions in HR and the time to 25, 50 and 75% reductions in RPP in group Bupi + Dex + Yoh were significantly increased than those in group Bupi (*P* <  0.001 for all comparisons, Table 1), but were no statistical difference with group Bupi + Dex (*P* > 0.05 for all comparisons, Table 1). HR was significantly lower in group Bupi than in the Bupi + Dex and Bupi + Dex + Yoh groups for all measurements from 2 to 6 min after the bupivacaine infusion (*P* <  0.05). RPP was significantly lower in group Bupi than that in the Bupi + Dex and Bupi+ Dex + Yoh groups for all measurements from 1 to 6 min after the bupivacaine infusion (*P* <  0.05). HRs and RPPs values showed no differences between the groups Bupi + Dex and Bupi + Dex + Yoh (*P* > 0.05).

### Cardiac tissue bupivacaine content

**Fig. 4:**
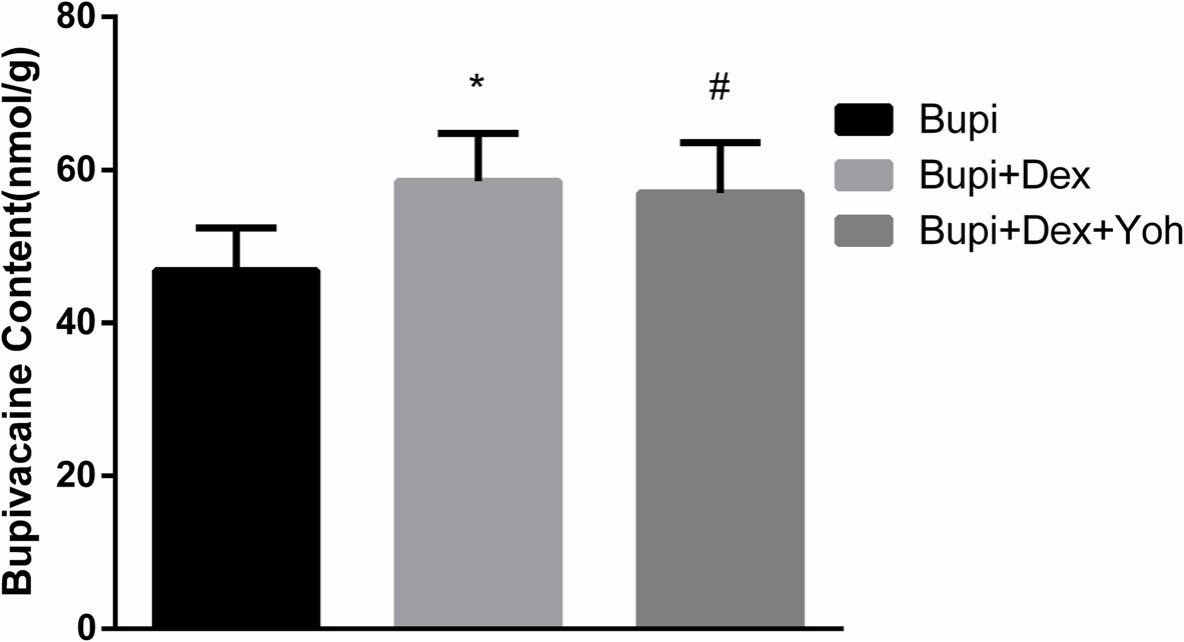
Bupivacaine content in cardiac tissue among the experiment groups (*n* = 8 in each group). Data were given as mean ± SD. ^*^P <  0.05, between group Bupi and group Bupi + Dex, ^#^*P* <  0.05, between group Bupi and group Bupi + Dex + Yoh. Con, control; Bupi, bupivacaine; Dex, dexmedetomidine; Yoh, yohimbine. Bupi + Dex vs. Bupi, *P* = 0.003, Bupi + Dex vs. Bupi + Dex + Yoh, *P* = 0.879, Bupi + Dex + Yoh vs. Bupi, *P* = 0.009

Cardiac tissue bupivacaine content after asystole in Bupi, Bupi + Dex and Bupi + Yoh + Dex groups was 46.8 ± 5.6 nmol/g, 58.5 ± 6.3 nmol/g and 57.0 ± 6.6 nmol/g respectively. Cardiac tissue bupivacaine content in the Bupi + Dex and Bupi + Dex + Yoh groups was higher than that in group Bupi (*P* = 0.003, *P* = 0.009, respectively, Fig. 4). There was no significant difference between the Bupi + Dex and Bupi + Dex + Yoh groups (*P* = 0.879).

## Discussion

Results of the present study showed as follows: (1) Dexmedetomidine had no effect on HRs and RPPs of the isolated rat hearts in group Dex. (2) Pretreatment with dexmedetomidine significantly increased the time to first arrhythmia and time to asystole in group Bupi + Dex. (3) The times to 25, 50 and 75% reductions in HR and RPP were significantly longer in the Bupi + Dex and Bupi + Dex + Yoh groups than in group Bupi. (4) Compared with group Bupi, dexmedetomidine significantly increased cardiac tissue bupivacaine content when asystole in goup Bupi + Dex and group Bupi + Dex + Yoh. (5) The benifit of dexmedetomidine in mitigating bupivacaine-induced cardiotoxicity could not be eliminated by yohimbine.

It was the first study to explore direct effects of dexmedetomidine on bupivacaine-induced cardiotoxicity in the isolated rat hearts. Compared with the model in vivo, this model could avoid the disturbance of internal environment and organ hypoxia. Based on our previous study [11, 12] and other study [13], we chose 50 μmol/L bupivacaine, 10 nmol/L dexmedetomidine and 1 μmol/L yohimbine as the optimal concentration.

Our results indicated that the cardiac function of the isolated hearts which perfused with KHB were stable throughout the experiment. Therefore, we can ignore the deterioration caused by the Langendorff heart model according the HR and RPP. Our study also showed that dexmedetomidine had no significant effect on HRs and the contractility of isolated rat hearts. In addition, Flacke et al. [14] also found in the isolated dog hearts that dexmedetomidine does not influence the heart function.

Hanci [8] proved that dexmedetomidine pretreatment delays occurrence of bupivacaine cardiotoxicity. Wistar-Albino male rats were pretreated with intravenous dexmedetomidine at a dose of 10 μg/kg in the bupivacaine-induced cardiotoxicity. The results showed that dexmedetomidine reduced the heart rates and mean arterial pressures and increased the time of first arrhythmia and asystole in the rats. However, Hanci speculated the mechanism may be related to dexmedetomidine’s sympatholytic properties and antiarrhythmogenic, without further investigation. Based on Hanci’s experiment, we suspected that dexmedetomidine could alleviate bupivacaine-induced cardiotoxicity by acting directly on the isolated heart.

In this study, we found the time to arrhythmia in the Bupi + Dex group was significantly longer than that in the Bupi group, indicating that dexmedetomidine exactly has anti-arrhythmic effects. Similarly, Chrysostomou et al. [10] also found that for the patients with perioperative atrial and junctional tachyarrhythmias, dexmedetomidine may have a potential therapeutic effect on controlling the HR or conversion to normal sinus rhythm. Therefore, we suspect the anti-arrhythmic effect is also involved in dexmedetomidine’s enhancing tolerance to bupivacaine cardiotoxicity in the isolated heart. However, the underlying mechanism of dexmedetomidine’s anti arrhythmogenic on isolated rat heart requires further investigation.

This study also showed that the myocardial concentrations of bupivacaine when asystole was significantly higher in group Bupi + Dex than in group Bupi, indicating dexmedetomidine may enhance the tolerance to bupivacaine-induced cardiotoxicity and increase the asystole concentrations of bupivacaine in the isolated rat hearts. This result is similar to previous study [8], which proved that dexmedetomidine enhanced the tolerance to bupivacaine-induced cardiotoxicity in rats.

Alpha 2 adrenoceptor is expressed and present in cardiomyocytes [15]. It is reported that the cardioprotective effect of dexmedetomidine on the ischemia/reperfusion injury in isolated rat hearts may be mediated via alpha 2 adrenoceptor [13]. Our research showed that yohimbine had no influence on the ability of dexmedetomidine on bupivacaine-induced cardiotoxicity in the isolated hearts. In the other words, dexmedetomidine enhanced the myocardial tolerance to bupivacaine-induced cardiotoxicity was not associated with alpha-2 adrenergic receptors. Accumulating evidence proves that dexmedetomidine can combine with other receptors or channels [16–18], and which suggests there may exist more mechanisms of dexmedetomidine’s protective effect on bupivacaine-induced cardiotoxicity.

The mechanism underlying bupivacaine-induced arrhythmia may be the blockade of cardiac voltage-gated sodium channels and L-type calcium channels, resulting in inhibition the production of the cardiac action potential [1]. The present study has demonstrated that bupivacaine causes arrhythmia or arrest. This result is similar to previous studies [19, 20]. Besides, our previous study [21] found that bupivacaine could inhibit the opening of the mitochondrial transition pore and disturb the balance of Ca^2+^ in mitochondria, which ultimately caused cardiomyocyte toxicity. Therefore, we speculate the mechanism that dexmedetomidine attenuates the cardiotoxicity of bupivacaine might also involve improved function of cardiac voltage-gated sodium channels and L-type calcium channels, and mitochondrial function.

Dexmedetomidine is increasingly being used as an adjuvant to local anesthetics in regional anesthesia procedures or for intraoperative sedation in clinical. Therefore, dexmedetomidine and bupivacaine will often be used together in patients. Our results showed that dexmedetomidine can increase the heart’s tolerance to bupivacaine and delay the onset of the cardiotoxicity of bupivacaine by acting directly on the heart. It suggests the use of dexmedetomidine may improve the safety use of bupivacaine in clinical, while more evidences like animal experiments and clinical studies need to be carried out.

### Limitations

Compared with Hanci’s study, the present study can directly reveal the effect of dexmedetomidine on the heart and the local mechanism of the ability of dexmedetomidine on bupivacaine-induced cardiotoxicity. However, it still has its limitations. First of all, we fail to explore the systemic effects of dexmedetomidine in vitro models. Besides, this study is unable to investigate the real-time concentration of myocardial bupivacaine in each group before asystole. We will design experiments to observe the impact of dexmedetomidine on the bupivacaine’s distribution in the myocardium in the further study.

## Conclusions

The results of the present study suggest that dexmedetomidine could enhances tolerance to bupivacaine cardiotoxicity on the isolated rat hearts, but the alpha 2 adrenoceptors were not involved in this process. Future studies should be concerned with the effects of dexmedetomidine on cardiac voltage-gated sodium channels and L-type calcium channels, and mitochondrial function.

## Data Availability

The datasets used and/or analyzed during the current study are available from the corresponding author on reasonable request.

## Abbreviations

Bupi: Bupivacaine
Con: Control
Dex: Dexmedetomidine
ECG: Electrocardiography
HR: Heart rate
KHB: Krebs-Henseleit buffer
RPP: Rate-pressure product
T_arrhythmia_: Time to the first arrhythmia
T_asystole_: Time to the asystole
Yoh: Yohimbine
